# The Relationship Between Selected Load-Velocity Profile Parameters and 50 m Front Crawl Swimming Performance

**DOI:** 10.3389/fphys.2021.625411

**Published:** 2021-02-19

**Authors:** Tomohiro Gonjo, Nikolai Njøs, Ola Eriksrud, Bjørn H. Olstad

**Affiliations:** Department of Physical Performance, Norwegian School of Sport Sciences, Oslo, Norway

**Keywords:** semi-tethered, race analysis, strength, velocity, technique, testing

## Abstract

The purpose of the present study was to establish relationships between sprint front crawl performance and a swimming load-velocity profile. Fourteen male national-level swimmers performed 50 m front crawl and semi-tethered swimming with three progressive loads. The 50 m performance was recorded with a multi-camera system, with which two-dimensional head displacement and the beginning of each arm-stroke motion were quantified. Forward velocity (V_50m_), stroke length (SL) and frequency (SF) were quantified for each cycle, and the mean value of all cycles, excluding the first and last cycles, was used for the analysis. From the semi-tethered swimming test, the mean velocity during three stroke cycles in mid-pool was calculated and plotted as a function of the external load, and a linear regression line expressing the relationship between the load and velocity was established for each swimmer. The intercepts between the established line and the axes of the plot were defined as theoretical maximum velocity (V_0_) and load (L_0_). Large to very large correlations were observed between V_50m_ and all variables derived from the load-velocity profiling; L_0_ (R = 0.632, *p* = 0.015), L_0_ normalized by body mass (R = 0.743, *p* = 0.002), V_0_ (R = 0.698, *p* = 0.006), and the slope (R = 0.541, *p* < 0.046). No significant relationships of SL and SL with V_50m_ and the load-velocity variables were observed, suggesting that each swimmer has his own strategy to achieve the highest swimming velocity. The findings suggest that load-velocity profiling can be used to assess swimming-specific strength and velocity capabilities related to sprint front crawl performance.

## Introduction

The shortest competitive swimming event is the 50 m freestyle, where the fastest swimmers finish in less than 21 s using the front crawl style. A 50 m short course freestyle performance (i.e., performed in a 25 m pool) is divided into five phases: the start, the first free-swimming phase, the turn, the second free-swimming phase, and the finish (Arellano et al., 2018a). Previous studies have clearly shown the importance of the start, turn, and finishing phases, highlighting their large contributions to overall performance (Mason and Cossor, 2000). Nevertheless, the free-swimming phases have the largest impact on the race outcome as swimmers spend the longest time (about 55% of the total race time) on these phases (Arellano et al., 2018b).

During front crawl free-swimming phases, performance is determined by the ability to produce and maintain the highest forward swimming velocity. This is achieved by maximizing and minimizing propulsive and resistive forces, respectively (Maglischo, 2003), and the propulsive force is linked to the ability to convert muscular to hydrodynamic forces. In other words, it is essential for swimmers to have both an ability to produce a large muscular force and proper technical skill to apply the force to the water (Maglischo, 2003). Therefore, it has been of great interest for researchers to investigate relationships between on-land strength measurements and swimming performance. It appears that correlations of on-land strength exercises with swimming race performance are greater in exercises similar to upper-limb joint movements during swimming (e.g., lat pulldown and swim bench) than those are not, such as bench press and squat (Morouco et al., 2011; Crowley et al., 2017). Nevertheless, one of the main critiques of the on-land strength exercises is that most of them lack specificity in terms of force production (applying forces to a solid object or the water). In other words, among the two important factors suggested earlier (muscular force production ability and conversion of the muscular to hydrodynamic forces), establishing relationships between on-land strength training and swimming performance can only show the importance of the former factor but not the latter one.

The fully tethered swimming approach mitigates the lack of force production specificity by measuring the force swimmers can produce while swimming at a fixed position. In this approach, swimmers are required to swim while being connected to a non-elastic wire (the other end of which is connected to a load-cell), and the maximum and mean forces in the whole-body swimming measured with this approach have shown large negative correlations with the finishing time of a short course 50 m front crawl (Loturco et al., 2016). However, during fully tethered swimming, swimmers are exposed to much larger water resistance against the hands and feet due to the whole-body not moving forward, which causes the technique during fully tethered swimming to be slightly different from the actual free-swimming condition (Samson et al., 2019).

Another approach that can be used to assess swimming-specific strength is semi-tethered swimming. Unlike fully tethered swimming, the swimmer moves forward in the water while being subjected to an external load. The forward motion induces relative streamwise water flows around the body, making the test more specific to the free-swimming condition than the fully tethered approach. This approach has been used to assess swimming power, which is calculated as the measured force multiplied by the swimming velocity (Shionoya et al., 1999; Dominguez-Castells et al., 2013a; Kimura et al., 2013). However, it is somewhat speculative to what extent this observed power is relevant to swimming performance. The force measured in semi-tethered swimming is the net force (the sum of the propulsive force, resistive force, and the force produced against the external load). From a macroscopic perspective, swimmers often swim with nearly a constant velocity that makes the sum of the mean propulsive and resistive forces during one stroke cycle almost zero. Therefore, the power calculated in semi-tethered swimming only considers the power against the external load and not the propulsive power produced by the swimmer.

Due to unsteady flow conditions around a swimming body, it is very complex to separately measure the propulsive and resistive forces acting on a swimmer (Samson et al., 2018). Hence, it is currently not possible to directly obtain propulsive power from semi-tethered swimming. However, this does not necessarily mean that semi-tethered swimming is impractical to assess the force production capability and swimming performance. Alternative use of semi-tethered swimming is to measure the velocity with different external loads to generate load-velocity profiles (Gonjo et al., 2020a; Olstad et al., 2020). The relationship between external loads and swimming velocity is highly linear [R^2^ ≥ 0.98 according to Gonjo et al. (2020a) and Olstad et al. (2020)], and thus, the maximum velocity at zero load (V_0_), maximum load at zero velocity (L_0_), L_0_ expressed as a percentage of body mass (rL_0_), and steepness of the slope for the load-velocity relationship can be estimated. Theoretically, V_0_ corresponds to the maximal free-swimming velocity, and the magnitude of the tethered force due to L_0_ should be equal to that of swimmer’s fully tethered swimming force. Therefore, these variables can potentially be used to assess both individual strength and velocity capabilities during swimming (Olstad et al., 2020). For example, a load-velocity profile with a large L_0_ but with a small V_0_ (and consequently a flat slope) implies that the swimmer is capable of applying a large force to the water but has a limited ability to use it effectively (or exposed to a large resistive force when moving forward) to produce a large velocity.

Given the potential of load-velocity profiling in swimming, it is important to assess which outcome parameters are related to swimming race performance. In butterfly, a previous study (Gonjo et al., 2020a) found significant correlations between free-swimming velocity during a 50 m race with V_0_ (r = 0.89) and L_0_ (r = 0.56), demonstrating load-velocity profiling as a useful method to assess 50 m butterfly swimming performance. However, these outcomes do not guarantee that the adequacy of the method can be generalized. For instance, it has been reported that the amount of external load affects the coordination between the left and right upper limbs in front crawl swimming (Dominguez-Castells and Arellano, 2012), which might cause a systematic error in predicting V_0_ and L_0_ in this particular stroke. However, the relationships been sprint performance and load-velocity profile in front crawl have not been explored yet. Front crawl swimming is the fastest swimming stroke, which means that it is the most appropriate stroke to be assessed for exploring human swimming ability. Indeed, to the best of the authors’ knowledge, front crawl is the most investigated stroke in human swimming research.

Considering the importance of front crawl swimming and the potential concern of using the semi-tethered swimming approach in front crawl, it would be highly beneficial to investigate the relationship between load-velocity profiling and front crawl performance. Therefore, the purpose of this study was to investigate relationships between load-velocity profile outcome parameters and 50 m front crawl swimming performance. It was hypothesized that both V_0_ and L_0_ would show strong relationships with 50 m front crawl swimming performance, but to a lesser extent than the reported butterfly results.

## Materials and Methods

### Experimental Approach to the Problem

#### Subjects

Fourteen male swimmers who qualified for the senior national championship in the 50 m front crawl (mean [SD]: age 19.9 [3.2] years, body height 187.1 [7.1] cm, body mass 80.8 [9.8] kg, personal best record 23.8 [0.8] s, FINA points 632 [48]) voluntarily participated in this study. The study was approved according to the Declaration of Helsinki by the local ethical committee and the National Data Protection Agency for research. All subjects gave their written informed consent prior to participation.

#### Design

A cross-sectional study design was used. Swimming performance was measured in a 25 m swimming pool (25 m × 12.5 m) with water and air temperatures of 28°C and 27°C, respectively. Anthropometric data were collected prior to testing in the water. The swimmers performed their own pre-competition warm-up (approximately 45 min) before the test, to be as close to a regular competition condition as possible. A 10–20 min active recovery followed the warm-up (Neiva et al., 2014), during which the subjects put on their competition swimsuits. The swimmers first performed one 50 m front crawl swim from a diving start, and after a 20 min rest (Neiva et al., 2014), three 25 m front crawl semi-tethered swims from a push-off start with maximal effort. The loads used in the three trials were 1, 5, and 9 kg, and swimmers had 5–10 min rest between each trial. This procedure has previously shown high reliability in assessing V_0_, L_0_, rL_0_, and the slope with intra-class correlation of ≥0.90 and coefficient of variation of ≤2.6% in a test-retest condition (Olstad et al., 2020). Each swimmer undertook all measurements within the same day.

A previous study (Olstad et al., 2020) has shown that assigning 9 kg load to swimmers imposes more than a 50% velocity reduction compared with a 1 kg load trial. Based on this observation and that a 50% velocity decrease has been suggested in multiple trial sprint testing with external loads (Cross et al., 2018b), the aforementioned absolute load protocol with a maximum load of 9 kg was deemed adequate. Furthermore, it has been reported that assigning more trials (i.e., five trials) in semi-tethered front crawl swimming does not change the outcomes of the load-velocity profiling compared with three trials (Olstad et al., 2020). Thus, based on these two rationales, the testing protocol employed in the current study was considered to be sufficient to establish a load-velocity profile in front crawl swimming.

#### Methodology

##### Anthropometric data collection

Anthropometric data were collected in accordance with the International Standards of Anthropometric Assessment (Stewart et al., 2011). Body height was measured with a stadiometer (Seca 213, Seca Deutschland, Hamburg, Germany), body mass with a weight scale (Seca 876, Seca Deutschland, Hamburg, Germany), and arm span (distance between the tips of the middle fingers) with a segmometer (HaB International Ltd., Warwickshire, United Kingdom).

##### 50 m front crawl performance

**FIGURE 1:**
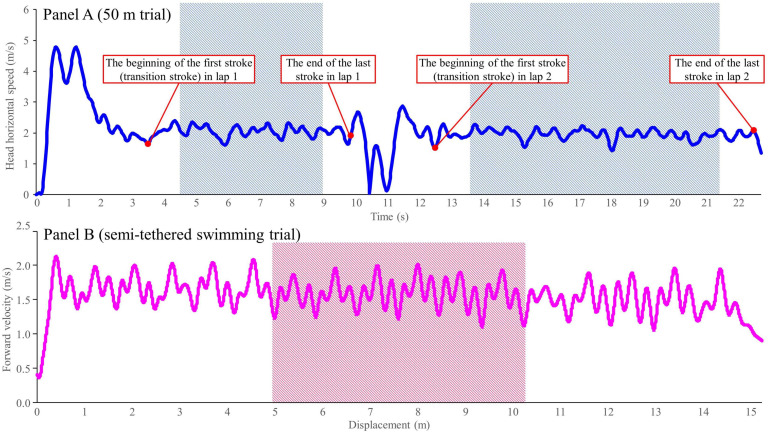
Examples of collected data from the 50 m front crawl (panel **A**) and semi-tethered swimming (panel **B**) trials. The shaded area shows the range of data used for the analysis.

The AIM race analysis system (AIMsys Sweden AB, Lund, Sweden) was used to record the 50 m front crawl race. The system included 11 cameras: five Axis Q3505-VE Mk II Network Cameras (Axis AB, Lund, Sweden) on land, and six Axis Q1635 Network Cameras (Axis AB, Lund, Sweden) placed behind windows below the water level. Five underwater and all above water cameras were located at the side of the pool with an approximate distance between each aligning camera of 5 m, which were used to record the motion of the swimmer throughout the race from the side view. One underwater camera was for recording a frontal view of the swimmers for feedback purpose, but not used for the analysis. An electronic timing system (Omega, Bienne, Switzerland) provided the finishing time for the 50 m front crawl (t_50m_). This system allows for automatic detection of two-dimensional head displacement and the timing of the beginning of each arm pull motion, based on an image processing technique and the side camera views obtained from the ten cameras, with a sampling frequency of 50 Hz. Detailed calibration algorithm for the system has been described in Haner et al. (2015). The mean velocity (V_50m_), stroke length (SL), and stroke frequency (SF) of each swimmer were calculated for all stroke cycles using the head displacement and stroke timing data obtained by the AIM system. For further data analysis, the mean values among all stroke cycles apart from the first and last cycle in each lap were used to minimize potential effects of transition stroke (from underwater to surface swimming) and turn preparation (Figure 1, panel A).

##### Load-velocity profiling

During semi-tethered swimming trials, the load was applied using 1080 Sprint, a robotic resistance device (1080 Motion, Lidingö, Sweden) incorporating a servo motor, 2000 RPM OMRON G5 Series Motor (OMRON Corporation, Kyoto, Japan) with a recording frequency of 333 Hz. The motor was connected to a composite fiber cord that was attached to the subject with a swim belt, S11875BLTa (NZ Manufacturing, OH, United States), and the velocity of the cord pulled out of the machine was recorded. The cord was attached to the swimmer’s back in order to avoid the wire disturbing the kicking motion. As the interest in the current study was mid-pool performance, the velocity data recording started and concluded at 5 m and 20 m from the wall, respectively (Olstad et al., 2020).

**FIGURE 2:**
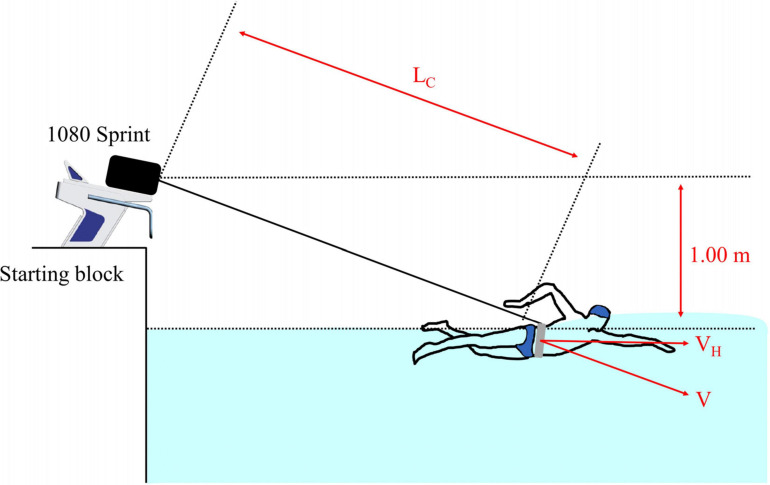
Testing setting for semi-tethered swimming trials. L_*C*_, V, and V_*H*_ show the length of the cord, velocity measured by the machine, and the horizontal component of the measured velocity, respectively.

**FIGURE 3:**
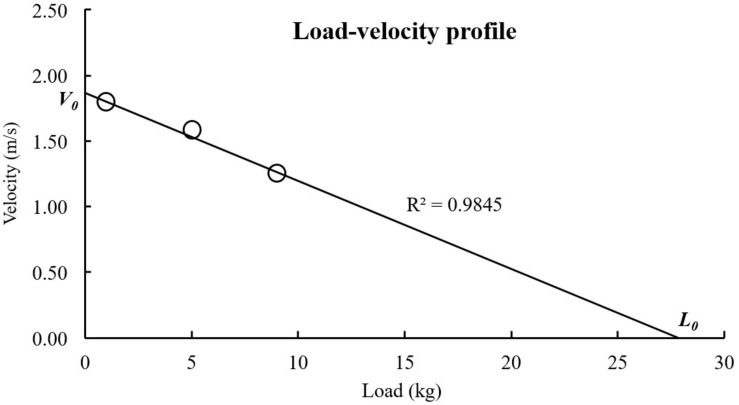
An example of a load-velocity profile (obtained from one subject tested in the current study).

Mean velocity achieved at a given load was calculated using the velocity curve of the cord generated by the 1080 Sprint software. For the velocity calculation, three consecutive stroke cycles at the middle of the pool were selected (Figure 1, panel B) to avoid any potential over- or under-estimation of the velocity (e.g., due to the push-off, fatigue, stroke adjustment for the touch) (Dominguez-Castells et al., 2013a). To assess the mean horizontal velocity, the following equation was used to adjust the velocity because the 1080 Sprint was positioned 1 m above the water level and the cord was not parallel to the water surface (Gonjo et al., 2020a).

$${\text{V}}_{\text{H}}=\text{V}\times \text{cos}\,\left[{\text{sin}}^{-1}\,\left(1.00/L_{\text{C}}\right)\right]$$

where V and V_*H*_ are the average velocities pre- and post-correction, respectively, 1.00 is the height (m) of the device (origin of the cord) from the water level, and L_*C*_ is the length (m) of the cord from the device to the swimmer (Figure 2). The average V_*H*_ during the three cycles were plotted as functions of the corresponding loads. A linear regression line was established for each load-velocity plot (Dominguez-Castells and Arellano, 2012). The coefficient of determination (R^2^) and theoretical maximal values of V_*H*_ (V_0_) and load (L_0_) were calculated using the regression line for each swimmer, and L_0_ was also expressed as a percentage of body mass (rL_0_). V_0_ represents the theoretical maximal velocity of each swimmer, whereas L_0_ represents the theoretical maximal load the swimmer can pull (without being towed backward) with front crawl swimming. The slope is the steepness of the linear regression line for the load-velocity relationship and was computed as Slope = –V_0_/L_0_. Figure 3 shows an example of a load-velocity profile for a single swimmer.

### Statistical Analyses

The Statistical Package for Social Sciences (SPSS) version 24 (IBM Corp., Armonk, NY, United States) was used to perform all statistical analyses, and the alpha level for significance was set to *p* < 0.05. Normal distribution of variables was assessed using the Shapiro-Wilk test. Confidence intervals were calculated for the assessed variables in accordance with Calder (1953). To show the level of agreement between the V_0_ and the V_50m_, a Bland-Altman plot was used (Bland and Altman, 1986). Pearson’s correlation coefficient was used to calculate all correlations for normally distributed data, while correlations between non-normally distributed data (age and arm span) and other variables were assessed using Spearman’s rank correlation coefficient. Correlation threshold values of 0.1, 0.3, 0.5, 0.7, and 0.9 were interpreted as small, moderate, large, very large, and extremely large correlations, respectively (Hopkins et al., 2009).

## Results

**FIGURE 4:**
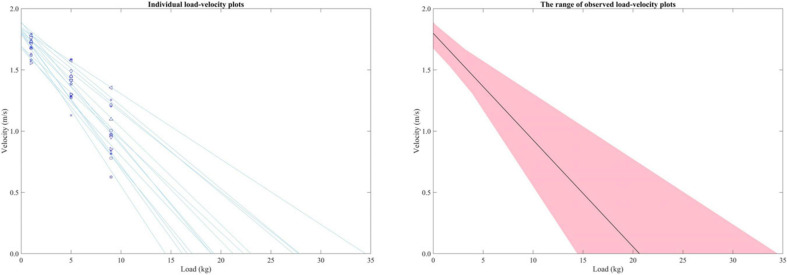
Individual load-velocity profiles obtained in the present study **(left panel)** and the inter-individual mean and the range of the load-velocity relationship **(right panel)**.

An overview of the distribution of load-velocity profiles for all subjects is presented in Figure 4. The left panel of the figure illustrates individual data, and the mean (solid line) and range (shaded area) in the right panel show the distribution of V_0_, L_0_ as well as the slope. Specifically, the L_0_ ranged from less than 15 kg to over 34 kg (mean L_0_ = 21.83 kg), while V_0_ ranged from less than 1.7 m/s to almost 1.9 m/s (mean V_0_ = 1.80 m/s). Numerical results for all variables obtained from the 50 m front crawl test and the load-velocity profiling are also shown in Table 1.

**TABLE 1:** Variables obtained from the load-velocity profiling and the 50 m front crawl tests.

	**L_0_ (kg)**	**rL_0_ (%)**	**V_0_ (m/s)**	**Slope (−m/s/kg)**	**R^2^**	**t_50m_ (s)**	**V_50m_ (m/s)**	**SF (cycles/min)**	**SL (m/cycle)**
Mean ± SD	21.83 ± 5.69	26.84 ± 0.81	1.80 ± 0.07	−0.09 ± 0.02	0.96 ± 0.03	24.26 ± 0.93	1.86 ± 0.06	55.76 ± 3.25	2.02 ± 0.12
CI_*upper95%*_	24.81	29.45	1.83	−0.08	0.98	24.75	1.89	57.46	2.08
CI_*lower95%*_	18.84	24.22	1.76	−0.10	0.95	23.77	1.83	54.05	1.96

*SD = standard deviation; CI_*upper95%*_ = upper bound of 95% confidential interval of Mean; CI_*lower95%*_ = lower bound of 95% confidential interval of Mean; L_0_ = estimated maximum load from the load-velocity slope; rL_0_ = estimated maximum load as a percentage of body mass; V_0_ = estimated maximum velocity from the load-velocity slope; Slope = steepness of load-velocity regression; R^2^ = coefficient of determination of the load-velocity regression line; t_50m_ = time for the 50 m front crawl; V_50m_ = mean free-swimming velocity during 50 m front crawl; SF = stroke frequency for the 50 m front crawl; SL = stroke length for the 50 m front crawl.*

Correlation coefficients between the obtained variables are presented in Table 2. Several significant correlations between variables from the 50 m front crawl and the load-velocity profile were observed: t_50_ had large negative correlations with L_0_ (-0.554, *p* = 0.000), rL_0_ (-0.677, *p* = 0.008), and V_0_ (-0.677, *p* = 0.008), and V_50m_ had large correlations with L_0_ (0.632, *p* = 0.015), V_0_ (0.698, *p* = 0.006) and the slope (0.541, *p* = 0.046), and very large correlations with rL_0_ (0.743, *p* = 0.002).

**TABLE 2:** Correlations between 50 m front crawl performance, load-velocity profile and anthropometric measurements.

	**SF**	**SL**	**V_50m_**	**L_0_**	**rL_0_**	**V_0_**	**Slope**	**Height**	**Body mass**	**Arm span**
t_50m_	−0.378 *0.183*	−0.179 *0.541*	−0.894** <*0.001*	−0.554* *0.040*	−0.677** *0.008*	−0.677** *0.008*	−0.468 *0.092*	−0.327 *0.254*	−0.115 *0.696*	−0.455 *0.102*
SF		−0.825** <*0.001*	0.353 *0.216*	−0.146 *0.617*	0.104 *0.725*	0.436 *0.119*	−0.22 *0.449*	−0.268 *0.354*	−0.467 *0.092*	−0.411 *0.144*
SL			0.223 *0.444*	0.498 *0.070*	0.301 *0.295*	0.002 *0.994*	0.505 *0.065*	0.526 *0.053*	0.568* *0.034*	0.714** *0.004*
V_50m_				0.632* *0.015*	0.743** *0.002*	0.698** *0.006*	0.541* *0.046*	0.426 *0.129*	0.167 *0.569*	0.481 *0.081*
L_0_					0.898** <*0.001*	0.335 *0.241*	0.951** <*0.001*	0.774** *0.001*	0.706** *0.005*	0.789** *0.001*
rL_0_						0.513 *0.061*	0.833** <*0.001*	0.527 *0.053*	0.330 *0.249*	0.710** *0.004*
V_0_							0.141 *0.630*	0.389 *0.169*	−0.128 *0.664*	0.253 *0.383*
Slope								0.692** *0.006*	0.745** *0.002*	0.763** *0.002*
Height									−0.068 *0.816*	0.569* *0.034*
Body mass										0.499 *0.069*

Numbers in plain font and *italics* show correlation coefficients and *p-value*, respectively. t_50m_ = time for the 50 m front crawl; SF = stroke frequency for the 50 m front crawl; SL = stroke length for the 50 m front crawl; V_50m_ = mean free-swimming velocity during 50 m front crawl; L_0_ = estimated maximum load from the load-velocity slope; rL_0_ = estimated maximum load as a percentage of body mass; V_0_ = estimated maximum velocity from the load-velocity slope; Slope = steepness of load-velocity regression; *p < 0.05; **p < 0.01.

**FIGURE 5:**
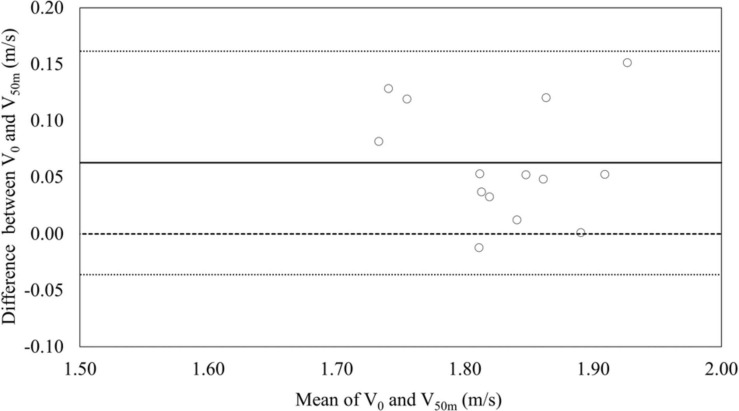
Bland-Altman plot showing the absolute agreement between maximum velocities estimated by the load-velocity profile and obtained from the race analysis.

Absolute agreement between V_0_ from the load-velocity profile and V_50m_ from the race analysis is shown in Figure 5. All points were within 1.96 SD levels, and the mean difference was around 0.06 (V_50m_ > V_0_).

## Discussion

The purpose of the current study was to assess the relationship between selected load-velocity profile outcome parameters (derived from three semi-tethered swimming trials) and 50 m front crawl swimming performance. The main findings were large to very large correlations between the 50 m front crawl parameters (t_50m_ and V_50m_) and the load-velocity profile parameters (L_0_, rL_0_, V_0_, and the slope), which suggest that variables such as L_0_, V_0_, and the slope from a load-velocity profile are good indicators of 50 m front crawl performance. The validity of the load-velocity relationship modeling is supported by the large average R^2^ values (0.96 ± 0.03), which are comparable to those found in other semi-tethered swimming studies (0.97–0.99) (Dominguez-Castells and Arellano, 2012; Gonjo et al., 2020a), and clearly show a linear relationship between velocity and load in semi-tethered swimming.

From the perspective that the L_0_ corresponds to a fully tethered swimming condition, it is logical that L_0_ is highly correlated with both V_50m_ and t_50m_, as significant relationships between maximum and mean fully tethered swimming force have been reported in the literature (Loturco et al., 2016). Given the extremely large correlation between the slope and L_0_ as well as the non-significant relationship between the slope and V_0_, it is very likely that the slope was strongly affected by L_0_, which means that the moderate-large association between the slope and V_50m_ can also be explained by the influence of L_0_. rL_0_ exhibited slightly stronger correlations with both V_50m_ and t_50m_ than L_0_. Assuming that the body mass of the subjects can be used as an indicator of their body size, the very large correlation is reasonable as a large body size does not only positively affect the propulsive force production but also increase the resistive force (Kjendlie and Stallman, 2011), meaning that a large velocity can be achieved with a small propulsive force production if the swimmer has to overcome a small resistive force.

A large correlation and agreement between V_50m_ and V_0_ were observed, however, the Bland-Altman plot (Figure 5) showed a systematic bias between V_50m_ (1.86 m/s) and V_0_ (1.80 m/s). A similar observation was reported in a recent study of load-velocity profiling (derived from semi-tethered swimming) and performance in butterfly swimming (Gonjo et al., 2020a), and as that study suggested, the explanation for the difference between V_50m_ and V_0_ might be due to the impact of the momentum of the start (and potentially the turn) and subsequent underwater swimming on the free-swimming phase during a race (Veiga and Roig, 2017). On the other hand, the effect of the non-free-swimming segments cannot be the only explanation for the observed difference between V_50m_ and V_0_. If that was the case, the magnitude of the difference should have been smaller in the front crawl as compared to the butterfly investigation (Gonjo et al., 2020a) since the effect of start and turns on the subsequent free-swimming phase is likely greater in butterfly than in front crawl (Veiga and Roig, 2017). However, the difference between V_50m_ and V_0_ in the current study was 3.53%, which is larger than that reported in the butterfly study (1.88%).

Another possible explanation for the smaller V_0_ compared with V_50m_ might be an increase in the index of coordination (IdC) due to the incremental external loading (Dominguez-Castells and Arellano, 2012). An increase in IdC indicates a greater overlap between the left and right upper-limb propulsive phases, which could potentially contribute to a high swimming velocity. Therefore, as the external load increased, the technique of swimmers might have changed and resulted in a greater contribution from the upper limbs to the propulsion as compared to lower external load conditions. This change would increase the slope (make it flatter) and lead to a smaller V_0_ in comparison to a hypothetical case with no IdC change in response to external loading. As IdC was not analyzed in the current study, the potential IdC change could be an interesting topic for future studies.

The small systematic bias between V_50m_ and V_0_ does not depreciate the large to very large correlations observed between outcomes from the load-velocity profiling and V_50m_ (and t_50m_). The results imply that V_0_ should not be used as a method to predict the “absolute” free-swimming velocity during a race, but it is still a good method to compare (e.g., between swimmers) or monitor (e.g., long-term development) the free-swimming velocity in 50 m front crawl. Overall, our initial hypothesis that both V_0_ and L_0_ would show strong relationships with 50 m front crawl swimming performance, but to a lesser extent than the reported butterfly results, was supported.

The non-significant correlation between L_0_ and V_0_ observed in the current study is consistent with a previous study on butterfly (Gonjo et al., 2020a). A lack of significance was also the case for the relationship between rL_0_ and V_0_. This means that some swimmers achieved both large V_0_ and L_0_, while others had a large V_0_ with a small L_0_ and vice versa. Given that L_0_ theoretically shows the fully tethered swimming ability, the lack of significant correlations likely reflects different strategies of the swimmers. More specifically, some swimmers rely on propulsive force production ability, but others minimize the resistive force to achieve a large V_0_. At a high loading condition, the ability to minimize the resistive force is not as important as producing a large propulsive force due to a low velocity. On the contrary, at a low loading condition, this ability is crucial due to a large forward velocity, meaning that swimmers who achieved V_0_ due to a steep slope might not have produced a great propulsive force, but might have had a proficient technique to minimize the resistive force. This explanation implies that the slope established in swimming load-velocity profiling could be an indicator of the resistive force (the steeper the slope, the greater the ability to minimize the resistance), which should be explored in future studies. This can also explain why there were no significant correlations between V_0_ and anthropometrics (height, weight and arm span) despite very large correlations of the anthropometric variables with L_0_, because a large body can contribute not only to great propulsive forces (and therefore L_0_) but also resistive forces that should largely affect V_0_ (Kjendlie and Stallman, 2011).

The different strategies for achieving a large V_0_ (maximizing the propulsion or minimizing the resistance) were also exhibited in the range of load-velocity profiles among the subjects (Figure 4), which shows a range of 92% of the mean for L_0_, but only a range of 12% for V_0_. However, it should also be noted that the load-velocity profiles established in the current study were based on the absolute load. Given that both the resistive and propulsive forces are affected by body size (Kjendlie and Stallman, 2011), the larger variability in L_0_ compared with V_0_ might be due to the variety of the anthropometry among the subjects. Therefore, obtaining swimming load-velocity profiles using the relative load (normalized by the body mass, height, or body surface area) might be useful in future studies. The use of relative load would probably not show different V_0_ and rL_0_ from the method used in the current study, but the interpretation of the slope might be more straightforward when it is assessed with profiles obtained with relative load and velocity outcomes. A potential benefit of using relative loads is also supported by the nearly significant correlation (0.513, *p* = 0.061) between V_0_ and rL_0_ (whereas V_0_ vs. L_0_ showed 0.335 and *p* = 0.241). Even though the relationship was not significant, the outcomes might imply that analyzing the relative load is more relevant to V_0_ as compared to absolute loads.

In the current study, L_0_ was very largely correlated with arm span, body mass and height. This was not surprising because the larger the body size, the larger the frontal surface area and underwater body volume that induce a greater resistive force (Gatta et al., 2015; Gonjo et al., 2020b), which consequently means that swimmers with a large body size should be capable of producing a large propulsive force to overcome the equivalent amount of resistive force (otherwise the swimmer cannot maintain a constant speed) (Kjendlie and Stallman, 2011). This result is in contrast to what has been reported previously in semi-tethered butterfly (Gonjo et al., 2020a). In that study, no significant correlations were observed between L_0_ and anthropometric variables. This suggests that anthropometric characteristics are more directly linked to propulsive force production in front crawl than in butterfly. This might be related to the complexity of butterfly swimming technique. For example, to produce large propulsion in butterfly, swimmers have to convert potential energy produced by the upper body to kinetic energy exerted by the lower body through undulatory motion (Sanders et al., 1995), which is not required in front crawl. Thus, the importance of technical skills relative to the anthropometry might be more evident in butterfly than in front crawl.

One limitation of the current study is a limited group of samples. This study focused on front crawl load-velocity profiling and its relationships with 50 m performance, but only in sprint male swimmers. Therefore, the results should not be generalized for other cohorts such as female and long-distance swimmers. In future studies, it would be particularly interesting to determine whether similar results are observed in long-distance swimmers. Despite the similarity in kinematic parameters (McCabe et al., 2011; McCabe and Sanders, 2012), long-distance swimmers are characterized by a larger percentage of type I muscle fibers than sprinters (Gerard et al., 1986), which might make a difference in the profiling. Another meaningful comparison would be between competitive long-distance swimmers and triathletes (or open water swimmers). Even though both groups are characterized as endurance athletes with similar neuromuscular demands, differences in the load-velocity profile might exist due to distinct kinematic characteristics between competitive swimmers and triathletes (Millet et al., 2002).

## Practical Applications

A practical advantage of the load-velocity profiling is that this method allows coaches and researches to monitor (e.g., long-term development) or compare (between swimmers) both propulsive force production ability and free-swimming velocity independent of start and turn effect.

Monitoring the development of a swimmer over time with load-velocity profiles will give insight into which factors (maximizing propulsion or minimizing resistance) causes changes in swimming performance. When a swimmer shows a large increase in V_0_ with an unchanged L_0_ after several months of training, this change suggests that this athlete has mostly improve his drag minimizing abilities, but showed little or no improvement in propulsive force production. Such an approach can also be used for a group of swimmers, which would contribute to monitor and improve training programs.

Comparing load-velocity profiles between athletes provides additional insight into performance. In the present study, one of the subjects showed V_0_ close to the group mean, nearly the lowest L_0_, and t_50m_ slightly slower than the group average, which implies that this swimmer has decent abilities to minimize drag but could still improve his propulsion production abilities compared to the other swimmers in the group. Another subject displayed the second largest L_0_, while his V_0_ and t_50m_ were only slightly above the group mean. In comparison to the other swimmers, this athlete might have a propulsive force advantage, but on the other hand, would probably benefit from focusing on minimizing drag.

Load-velocity profiling may also be used for establishing requirements for performance at different levels for the 50 m front crawl, e.g., between national and international levels of performance. FINA points are a common way to differentiate between performance levels. While this approach is good at standardizing the total race performance levels between different events, it cannot explain free-swimming performance determinants. Load-velocity profiling could provide practitioners and coaches with feedback on areas of potential improvement pertaining to propulsion and drag.

## Conclusion

The current study found large to very large significant relationships between all parameters obtained from load-velocity profiling and 50 m front crawl performance parameters in swimmers competing at the national elite level. In particular, V_50m_ exhibited the largest correlation with rL_0_ (R = 0.743), followed by V_0_ (R = 0.698), L_0_ (R = 0.632), and the slope (R = 0.541). The findings suggest that load-velocity profiling can be used to assess propulsive force production and velocity capabilities related to front crawl sprint performance.

## Data Availability Statement

The original contributions presented in the study are included in the article and the Supplementary Material. Further inquiries can be directed to the corresponding author.

## Ethics Statement

The studies involving human participants were reviewed and approved by Norwegian School of Sport Sciences. The patients/participants provided their written informed consent to participate in this study.

## Author Contributions

BHO and OE developed the concept of the study. BHO recruited the study subjects. TG, NN, and BHO conducted data collection. TG, OE, and BHO treated data and ran primary analyses, which NN summarized. TG and NN worked on the draft of the manuscript. All authors contributed to editing the article and approved its final form.

## Conflict of Interest

OE is a shareholder in 1080 Motion AB. The remaining authors declare that the research was conducted in the absence of any commercial or financial relationships that could be construed as a potential conflict of interest.

## Supplementary Material

Supplementary Figure 1 Individual load-velocity profiles for all subjects.
