# Supplementary figures and images for: The Relationship Between Selected Load-Velocity Profile Parameters and 50 m Front Crawl Swimming Performance
Source: Front Physiol. 2021 Feb 19;12:625411. doi: 10.3389/fphys.2021.625411 (PMC7933527; doi:10.3389/fphys.2021.625411)

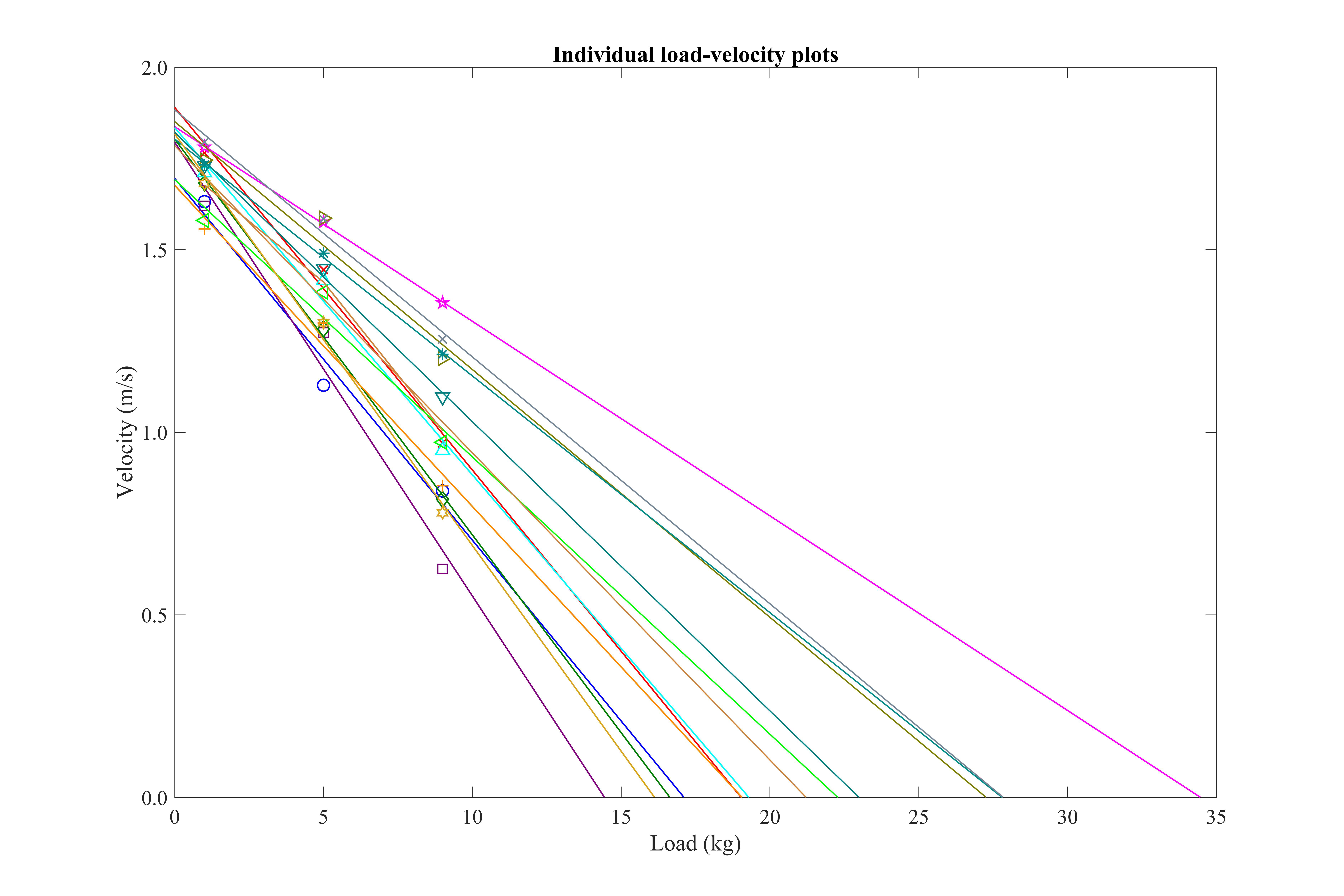

Supplement: Supplementary Figure 1 — Individual load-velocity profiles for all subjects. [file Image_1.JPEG]
